# Long‐Term Effects of a Web‐Based Exercise Programme for People With Intellectual Disabilities

**DOI:** 10.1111/jir.70082

**Published:** 2026-01-19

**Authors:** Sanna Fjellström, Nicole Stuffler, Erik P. Andersson, Anna Nordström, Eva Flygare Wallén, Elisabeth Hansen, Marie Lund Ohlsson

**Affiliations:** ^1^ Department of Health Sciences, Swedish Winter Sport Research Centre Mid Sweden University Östersund Sweden; ^2^ School of Sports Science UiT The Arctic University of Norway Tromsø Norway; ^3^ Department of Medical Sciences Uppsala University Uppsala Sweden; ^4^ Department of Neurobiology, Care Sciences and Society (NVS) Division of Occupational Therapy Karolinska Institutet (KI) Stockholm Sweden; ^5^ Health and Social Care Administration Municipality of Östersund Östersund Sweden; ^6^ The Faculty of Education and Arts Nord University Bodø Norway; ^7^ Department of Physiology, Nutrition and Biomechanics The Swedish School of Health and Sport Sciences (GIH) Stockholm Sweden

**Keywords:** developmental disability, digital tools, e‐health, physical activity

## Abstract

**Background:**

Physical activity is essential for preventing noncommunicable diseases and improving health parameters. However, individuals with intellectual disabilities often struggle to meet recommended activity levels. Sustainable solutions and long‐term follow‐up are crucial for evaluating intervention efficacy.

**Methods:**

This mixed‐method longitudinal follow‐up study examines the effects and experiences of a 12‐week web‐based exercise programme on individuals with intellectual disabilities (ID). Body composition, physical activity levels and waist circumference were measured before and after the 12‐week intervention period as well as 12 months after the end of the intervention period (i.e., long‐term follow‐up). Experiences were analysed using semistructured interviews. In the data analysis, repeated measures ANOVA with Bonferroni correction was utilised to investigate changes over time.

**Results:**

No significant changes were observed after 12 months, but there were effects on postintervention compared with preintervention on waist circumference. Some participants reported experiencing health benefits, which contributed to motivation, while others lacked motivation and were unaware that they could continue to exercise.

**Conclusions:**

While improvements were noted post‐intervention, sustaining these gains proved challenging during long‐term follow‐up. This study highlights the potential of web‐based exercise programmes to support individuals with ID in increasing physical activity levels. However, the findings also underscore the need for more tailored and sustainable interventions, including structured support and ongoing engagement strategies, to enable lasting health behaviour change over time.

**Abbreviations**
BMI: body mass index
ICF: International Classification of Functioning, Disability and Health
ID: intellectual disabilities
IPAQ‐SF: International Physical Activity Questionnaire—Short Form
PA: physical activity
PAD model: The Physical Activity for People with a Disability model
WHO: World Health Organization

## 1 Background

Physical inactivity is the fourth leading risk factor for premature death (Dumith et al. 2011; Katzmarzyk et al. 2022). Many studies have shown promising results in preventing and improving noncommunicable diseases by increasing physical activity (PA) (Lee et al. 2012; Centers for Disease Control and Prevention 2020). However, long‐lasting PA interventions for people with intellectual disabilities (ID) remain less understood. Many studies do not report physical activity maintenance, and previous systematic reviews have shown that long‐term follow‐ups often do not find significant results (Castro et al. 2018).

People with ID are a vulnerable group in society that seldom or never reach the recommendations of PA (Doody and Doody 2012; Jacinto et al. 2021). In 2020, the World Health Organization (WHO) updated its recommendations for PA with a special announcement for people with disabilities (Bull et al. 2020). The recommendations for people with ID are the same as for the general population, namely, 150–300 min per week at moderate intensity. The background to the updated recommendations is the target group's alarming health aspect, including a higher risk of developing cardiovascular disease, higher blood pressure, BMI and prevalence of overweight and obesity (Doody and Doody 2012; Emerson et al. 2016). Additionally, people with ID are more likely to develop mental disorders (Yang et al. 2022). Regular physical activity can play a pivotal role in preventing cardiovascular diseases and mental health problems, consequently reducing the risk of mortality and morbidity (Elinder et al. 2010; Ross et al. 2020; Jacinto et al. 2023; Jacob et al. 2023).

Approximately 1%–3% of the population has an intellectual disability, defined as a limitation in intellectual ability and adaptive skills (Schalock et al. 2021). Several barriers prevent people with ID from reaching PA recommendations, such as transport issues, the need for support persons, economic aspects and nonadapted exercise facilities (Jacinto et al. 2021; Bossink et al. 2017). However, there are many possibilities to enhance PA in the ID target group, such as structured planning, help from a support person and commitment to follow the recommendations for PA (Jacinto et al. 2021). The Physical Activity for People with a Disability (PAD) model, which includes personal and environmental factors and behavioural strategies, can help identify key strategies to improve long‐term physical activity adherence (van der Ploeg et al. 2004). The International Classification of Functioning, Disability and Health (ICF) model (World Health Organisation 2001) is integrated into the PAD model as a framework for understanding the complex interplay between an individual's health condition, environmental factors and personal factors. Key elements include creating an inclusive, accessible and supportive environment, providing appropriate guidance and encouragement by support persons, and ensuring activities are enjoyable and feasible. Additionally, providing positive feedback and tracking progress to maintain motivation can improve self‐efficacy and thus improve compliance with PA.

Digital solutions have demonstrated success in enhancing PA levels among various target groups (Ballin et al. 2020; Vikberg et al. 2019). However, there is a gap in research regarding the exploration of web‐based exercise programmes specifically tailored for people with ID. Previous research has explored various digital approaches to promote physical activity among people with ID. For example, VR‐based exercise programmes have shown positive effects on resting heart rate (Lotan et al. 2010), but their high cost limits accessibility. More recent reviews (e.g., Park et al. 2024; Van Biesen et al. 2024) have examined web‐based interventions, including active video games, social media platforms and supported self‐management strategies. However, these studies often targeted children or focused on educational content rather than structured exercise programmes for adults. Positive outcomes were typically linked to support from staff or family. Despite differences in format and target groups, these reviews underscore both the challenges and potential of digital tools to promote physical activity in this population.

In a prior investigation, we examined the impact of a web‐based exercise programme tailored for individuals with ID (Fjellstrom et al. 2022). Based on the PAD model, the intervention created a supportive environment with trained staff to enhance PA maintenance. Digital solutions can prevent some barriers to PA and are time‐efficient for both staff and the target group. However, the long‐term effects of such exercise programmes have not yet been analysed. Maintaining regular PA is essential for improving cardiovascular health and enhancing overall physical fitness. Sustaining these health benefits over the long term can significantly help prevent noncommunicable diseases such as obesity, diabetes and heart disease. Additionally, for individuals with ID, who face an elevated risk of mental health challenges, sustaining regular physical activity can yield substantial psychological benefits. Building on a previously conducted 12‐week web‐based exercise intervention, the present study aimed to evaluate its long‐term effects on health parameters 12 months after the programme's completion.

### 1.1 Research Questions

What are the observed effects 1 year after a 12‐week web‐based exercise intervention on physical activity levels, weight, fat mass, lean mass and waist circumference? Additionally, how many participants continued exercising post‐intervention, and what were their long‐term experiences with such exercise?

## 2 Methods

### 2.1 Study Design

**FIGURE 1:**
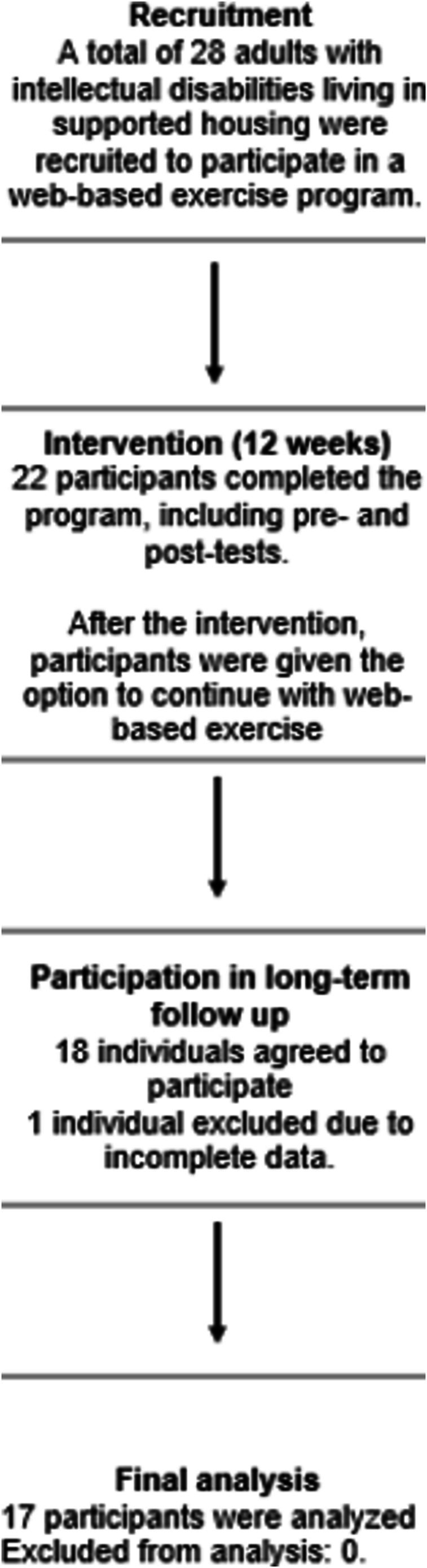
Flowchart illustrating the intervention process and participant progression throughout the study.

The present study is a longitudinal follow‐up study following a 12‐week web‐based exercise intervention with pre‐and post‐tests (Figure 1). A mixed‐method approach was conducted to evaluate both the quantitative effects and qualitative experiences of the exercise programme.

### 2.2 Participants

The inclusion criterion for participation in the present study was prior participation in the intervention study by Fjellstrom et al. (2022). In that study, participants were required to have an ID (defined as an IQ below 70), be able to follow instructions delivered via screen, have adequate vision and not require walking aids. All participants from the initial study (*n* = 22) were invited to partake in the long‐term (12‐month) follow‐up. At the start of the intervention study, participants were informed that a follow‐up would be conducted both after 12 weeks and after 1 year. However, understanding and remembering what a year entails can be challenging (Carlson 2013). Consequently, a new consent form was developed after the 12‐month period had elapsed, and participants were subsequently asked again if they wished to continue their involvement in the study. Of the 22 participants, 18 agreed to participate, with a mean age of 37.4 ± 9.7 years, and 46.7% were females.

### 2.3 Intervention

Participants from the intervention study (Fjellstrom et al. 2022) were instructed to follow a web‐based exercise programme three times a week, with each session lasting 50 min over 12 weeks. The exercise programme was provided through the commercial platform MyMOWO (https://www.mymowo.com, Virtual Gym Sweden AG, Sweden). The programme adhered to the WHO's recommendation of 150 min of physical activity at a moderate intensity level per week.

The exercise programme was specifically adapted for individuals with ID in collaboration with a special education teacher, a physiotherapist and an instructor from the commercial platform. Each session lasted 50 min, and participants could choose to complete the full session at once or split it into two shorter segments (i.e., 2 × 25 min). The programme included simplified instructions, longer exercise durations, hydration reminders and a visible timer. Training sessions were delivered via video and could be performed individually or in groups, either at home or in community facilities.

To further promote adherence, researchers held two online group meetings during the intervention (at Weeks 4 and 8) to identify and address potential challenges and maintain engagement. A more detailed description of the intervention can be found in Fjellstrom et al. (2022).

Following the 12‐week intervention period, the staff were informed that the participants with ID could choose to continue their membership with MyMOWO. However, no information concerning the 12‐month long‐term follow‐up was provided after the end of the intervention.

The participants' prior experiences with physical activity were explored through interviews conducted before and after the 12‐week exercise programme (Hansen et al. 2023, in press). Most participants (13 out of 17) initially described sedentary lifestyles and associated exercise with negative emotions, discomfort or previous injuries, particularly involving the knees and back. Despite these ambivalences, all participants acknowledged the health benefits of engaging in regular physical activity.

### 2.4 Data Collection

#### 2.4.1 Effects of the Web‐Based Intervention

The long‐term follow‐up effects were assessed by measuring body composition, waist circumference and PA level. Body composition was measured using a Body Composition Analyser (InBody 270, Seoul, South Korea), which utilised Bioelectrical Impedance Analysis (BIA) (2016 InBody Co. Ltd). Measurements were made at the same time of the day for pre‐, post‐ and long‐term follow‐up. Participants were instructed to step onto the scale with light clothes, bare feet and with their feet aligned with the foot electrodes. They were instructed to hold their hands on the handles with their thumbs placed on the oval electrodes and their arms stretched out from their body. Fat mass and lean body mass were measured in kilograms. Waist circumference was measured in centimetres, midway between the lowest rib and the iliac crest with a measuring tape (Ross et al. 2020).

The PA level was measured using the short form of the International Physical Activity Questionnaire (IPAQ‐SF), which assesses PA levels over the last 7 days (Kurtze et al. 2008). The IPAQ‐SF has shown good correlations with objective measures and is supported by evidence for use in populations both with and without ID (McKeon et al. 2013; Kurtze et al. 2008; Cleland et al. 2018).

The IPAQ consists of questions addressing the PA level during a week, collecting days, hours and minutes of vigorous PA, moderate PA, walking and sitting time. The measures result in the metabolic equivalent of task (MET) (for details, see Table 1) and three levels of PA (low, moderate and high) were reported according to the IPAQ protocol (24) (http://www.ipaq.ki.se/).

**TABLE 1:** Examples of MET values and how to convert activity‐time and level to MET‐minutes when using IPAQ‐SF.

Activity	MET value	Time (min)	Total MET minutes^a^
Walking	3.3	60 (ex)	198
Moderate intensity	4.0	30 (ex)	120
Vigorous intensity	8.0	45 (ex)	360
Total			678

^a^ MET = metabolic equivalent of task.

Variables used in this study are moderate‐vigorous PA (MVPA), walking time and total MET minutes. To ascertain whether individuals have met the recommendations for physical activity, MET minutes were calculated for MVPA. They need to achieve at least 600 MET‐minutes on moderate to high levels of PA, which can be attained in various ways—slightly more when engaging in moderate intensity and slightly less in vigorous intensity. The sum provides a total value for MVPA. Additionally, the 1‐year follow‐up included two questions about whether they had continued with the web‐based exercise, other PA or not continued at all with PA, and whether someone had asked if they wanted to continue with the web‐based exercise programme.

#### 2.4.2 Experiences of the Web‐Based Exercise

A semistructured questionnaire with both closed and open questions was conducted to capture the long‐term experiences of the web‐based exercise programme. The questions focused on experiences and factors influencing compliance with physical activity participation. Two authors conducted interviews with the participants in their respective apartments within the group living home. For some of the participants, a support staff was available to assist and confirm the participant's responses at the participant's request.

### 2.5 Data Analysis

Descriptive statistics were presented as means with standard deviation (SD) or, when appropriate, as median and interquartile range (IQR). The Shapiro–Wilk test for normality was used to assess whether data were normally distributed. For normally distributed data, repeated measures ANOVA with Bonferroni correction was conducted to analyse the effects of the exercise programme; for nonnormally distributed data, the Friedman test was used. The analysis included data on body composition, waist circumference and physical activity level at pre‐test, post‐test and long‐term follow‐up. To analyse the binary data representing whether participants met the physical activity recommendations at different time points (pre‐test, post‐test and long‐term follow‐up), we employed Cochran's *Q* test. The level of statistical significance was set at *p* < 0.05. Data analysis was performed using IBM SPSS Statistics for Windows version 27 (IBM Corporation, Armonk, NY, USA).

The interviews were analysed using a qualitative content analysis (Graneheim and Lundman 2004), focusing on the manifest content. The transcribed interviews were read through multiple times by two of the authors to gain a better picture of the entirety of all data gathered. Meaning units were identified by the authors separately and then coded and sorted into categories and subcategories. The authors compared the outcomes and discussed the results until a consensus was reached (Graneheim et al. 2017). The PAD model (van der Ploeg et al. 2004) was used as a theoretical framework to structure the analysis. The model helps to examine how individual, environmental and activity‐related factors, as well as participants' stages in the change process, affect exercise behaviours. The qualitative data provided insights into the participants' long‐term experiences of the web‐based exercise programme.

## 3 Results

Of the 18 participants, data from 17 were analysed due to missing data on the post‐test for one individual. All 17 participants were a part of both the qualitative and quantitative parts of the analysis.

### 3.1 Effects on Body Composition and Waist Circumference

The results are presented in Table 2. There was a significant main effect for BMI, Wilks' *λ* = 0.65, *F* (2.32) = 4, *p* = 0.04, and a significant main effect for waist circumference, Wilks' *λ* = 0.62, *F*(2.32) = 3.3, *p* = 0.03. Post hoc analysis with Bonferroni correction showed that waist circumference significantly decreased between pre‐ and post‐intervention (*p* = 0.03). No other significant results were observed.

**TABLE 2:** Body composition and waist circumference at pre‐ and post‐intervention, as well as the long‐term follow‐up for 12 months that started directly after the intervention (*n* = 17).

	Pre‐intervention (Mean ± SD)	Post‐intervention (Mean ± SD)	Long‐term follow‐up (Mean ± SD)	*p*	Effect size (partial *η* ^2^)
Body mass (kg)	82.2 ± 20.4	81.1 ± 20.6	82.6 ± 21.8	0.1	0.26
BMI (kg/m^2^)	32.6 ± 8.1	32.1 ± 8.3	33 ± 8.4	0.04	0.36
Lean mass (kg)	28.1 ± 7	27.8 ± 6.6	27.8 ± 6.6	0.9	0.1
Fat mass (kg)	31.5 ± 15.5	31 ± 16.9	32.4 ± 17.5	0.2	0.2
Fat mass (%)	36.9 ± 13.6	36.4 ± 14.8	37.6 ± 13.8	0.3	0.1
Waist circumference (cm)	102.8 ± 19.2	99.2 ± 17.6	102 ± 19	0.03	0.4

### 3.2 Effects on PA Level

**FIGURE 2:**
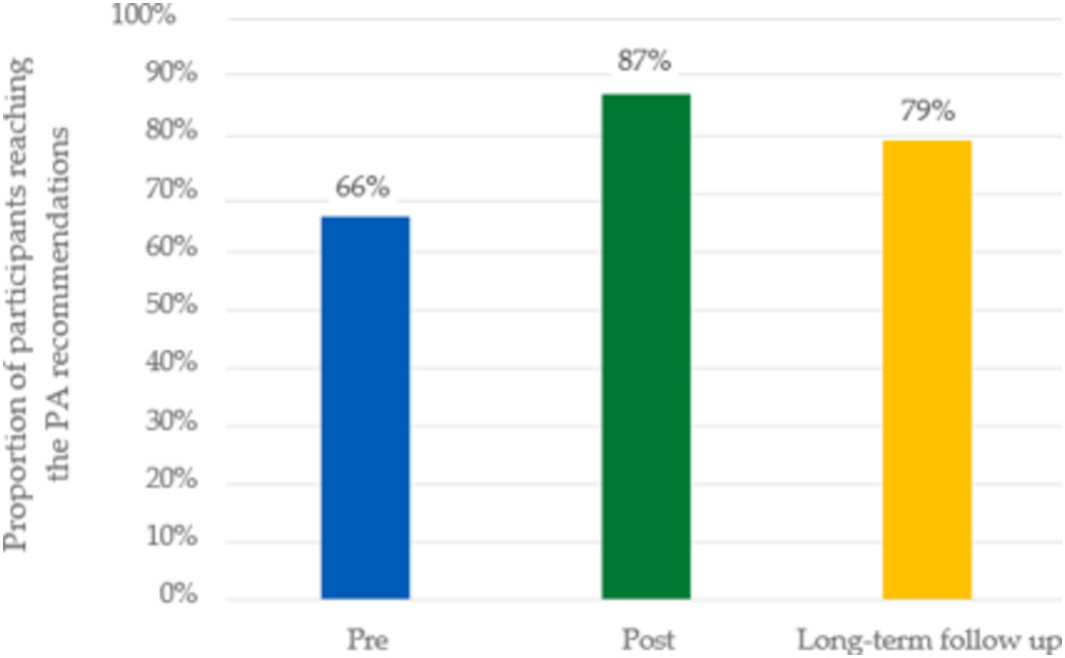
The proportion of participants meeting the recommendations of physical activity (PA) according to IPAQ‐SF at pre‐ and post‐intervention as well as 12 months after the intervention, that is, ‘long‐term follow‐up’.

The results are presented in Table 3. No significant results were reported when comparing the MET minutes from the three different measurement periods. When converting the MET minutes to PA levels, the number of participants in per cent meeting the recommendations of 600 MET‐min of MVPA (Figure 2) also did not show any significant differences.

**TABLE 3:** Physical activity levels at pre‐ and post‐intervention (3 weeks), as well as the long‐term follow‐up for 12 months that started directly after the intervention (*n* = 13).

IPAQ‐SF (MET‐min)	Pre‐intervention Median (IQR)	Post‐intervention Median (IQR)	Long‐term follow‐up Median (IQR)	*p*	Effect size (partial *η* ^2^)
Walking	495	891	660	0.9	0.01
	(99–1386)	(197–990)	(247–1485)		
MVPA	360	400	480	0.5	0.1
	(0–2208)	(100–2640)	(300–945)		
Total	942	1070	660	0.6	0.08
	(347–3024)	(897–3531)	(247–1485)		

Abbreviations: IPAQ‐SF: International Physical Activity Questionnaire—Short Form. IQR: interquartile range. MET‐min: metabolic equivalent of task minutes.

The results of the sustained PA engagement are presented in Figure 2. The question concerning how many continued with either web‐based PA, other PA or no PA at all revealed that participants were evenly distributed between web‐based PA and other forms of PA, with fewer individuals not continuing any form of physical activity.

**FIGURE 3:**
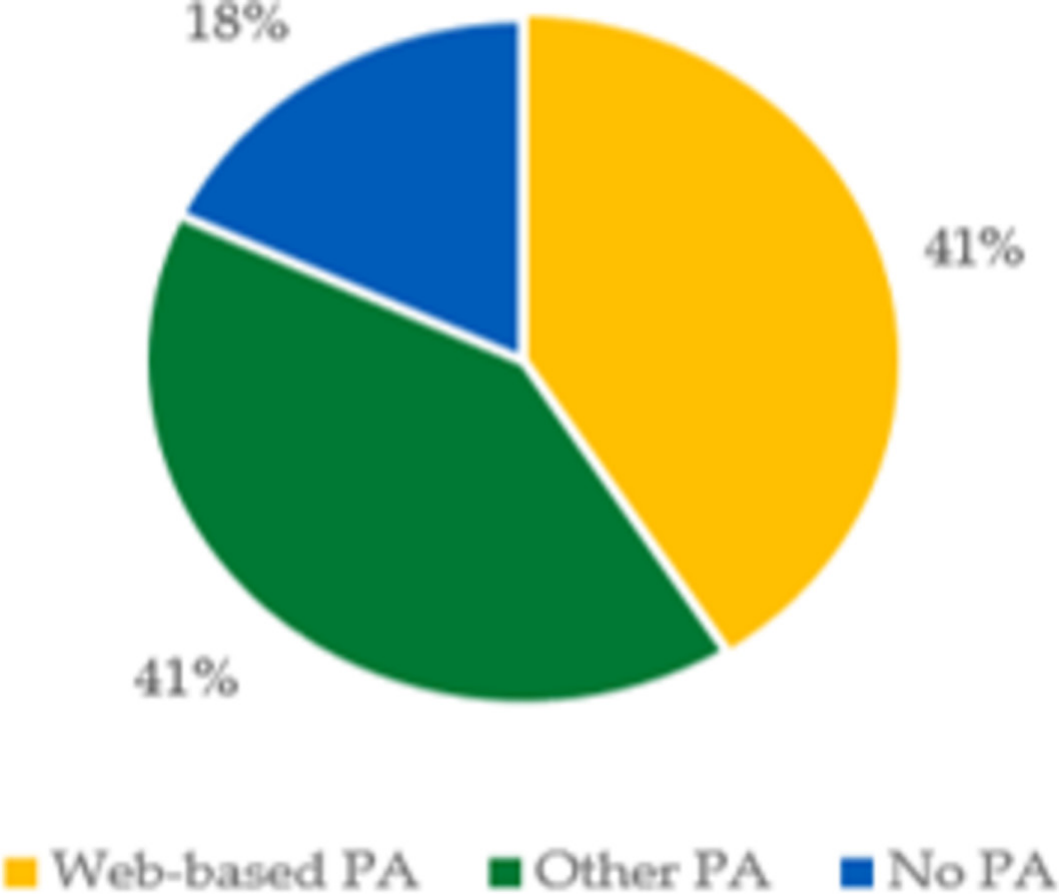
Distribution of participants continuing web‐based PA, Other forms of PA or no PA. Responses of how and if the participants continued or did not continue with PA 12 months after the intervention. The results are presented in three groups: those who continued with web‐based exercise, those who continued with other types of PA, those who did and those who did not continue with PA.

As a follow‐up to the responses presented in Figure 3, participants were asked whether someone had inquired if they wanted to continue with the web‐based exercise programme. These results are shown in Table 4. Participants who did not continue with any physical activity were not asked this follow‐up question.

**TABLE 4:** The number of people who were asked if they wanted to continue with the web‐based exercise programme was categorised by whether/how they continued training.

Have you continued with PA?	Yes—web‐based PA (*n* = 7)	Yes—other PA (7)	No—not at all (3)
Did someone ask if you wanted to continue with the web‐based exercise?	Yes (*n* = 4) No (*n* = 3)	Yes (*n* = 5) No (*n* = 2)	Yes (*n* = 0) No (*n* = 3)

### 3.3 Factors Behind PA Engagement

The qualitative content analysis resulted in four categories and eight subcategories (Table 5). Representative quotes can be found in the main text following the descriptions of the categories and subcategories.

**TABLE 5:** Categories (in bold text) and subcategories.

Personal factors	Environmental factors	Activity‐related factors	Stages of change
Health and well‐being	Social interaction	Design of exercise programmes	Varied levels of commitment to maintaining physical activity
Motivation and interest	Technical and practical barriers	Alternative forms of exercise	
	Importance of support staff		

#### 3.3.1 Personal Factors

Many participants continued with web‐based exercise because they felt improvements in their physical and mental health.

I feel great. So I have neither heart pain nor … Yes, everything feels superb. (P1)

Some participants also mentioned that losing weight or preventing gaining weight was one reason to keep on exercising and taking care of the body.

It's because I wanted to be active and I didn't want to gain weight. (P10)

From a health perspective, I need to lose weight. On both my mother's and father's side, there are heart problems, and I don't want to end up that way. My father died due to a heart problem. (P17)

Additionally, some participants described feeling more energised and stronger, which motivated them to continue, while others felt they simply lacked the motivation and therefore stopped web‐based exercise.

I lose interest very quickly, and exercise is one thing I wish I was more interested in, but unfortunately, I'm not. (P4)

For three participants, pain in their bodies was a barrier to not continuing exercising.

#### 3.3.2 Environmental Factors

The social aspect of exercising motivated some participants to continue.

It's mostly because it's fun. And we usually all exercise together, so it becomes a fun activity to do with your neighbors. (P8)

Some participants highlighted that the exercise programme being free of charge and eliminating the need for travel were significant benefits that motivated them to continue. However, technical issues and lack of space were common barriers to participating in the web‐based exercise programme.

It's more the lack of space than anything else. I have the computer in the bedroom, and there's not much room between the desk and the bed. (P13)

The interviews revealed that there was a lack of information, and participants did not know they could continue with the web‐based exercise. One participant could not explain why he/she did not continue with the web‐ based PA, but when asked if he/she knew that it still was possible to do the web‐based PA the participant was surprised and responded that he/she wanted to continue. Another participant brought up that he/she wanted to continue but did not know whether one was supposed to continue or not.

I Do you know you can continue with the web‐based workout on the computer?

P I can?

I Yes.

P Then I want to continue with it. (P17)

#### 3.3.3 Activity‐Related Factors

For some participants, it was important that the training was varied and engaging. Some participants thought the exercises were too difficult, while others enjoyed the challenges the programme gave. The difficulties that one participant mentioned were problems viewing the screen due to visual impairment, while some others mentioned that the exercises were too complicated. Another person preferred to exercise in a group setting at a fitness facility. Some participants preferred other forms of physical activity, such as walking, which led them to stop web‐based exercise.

I think it's nice to get out and get some fresh air. (P3)

#### 3.3.4 Stages of Change

Based on the stages of change model, answers from the interviews seem to reflect that the participants were spread over all five different steps of behaviour change. Some participants had no intention of continuing web‐based exercise, often due to a lack of interest or motivation. However, some participants were considering participating but were still uncertain.

It feels like I don't have time; there's so much else going on all the time. (P12)

I feel better, not as tired. That's good. But I also work, so I do not have the energy to exercise every day. (P15)

Some participants were ready to start or continue the web‐based exercise programme and sought information or support. Participants in the fourth and fifth phases actively engaged in the web‐based exercise programme or other forms of exercise, incorporating it into their daily routines and successfully maintaining their web‐based exercise regimen.

I work out on Wednesdays, Saturdays, and Sundays (…) because I feel good in my body. (P9)

## 4 Discussion

The present study reveals the effects of a 12‐month long‐term follow‐up after conducting a 12‐week web‐based exercise intervention for people with ID.

To the best of the authors' knowledge, this study is the first to present long‐term results from a web‐based exercise programme for this specific target group. The main results were that no significant changes were observed after 12 months, but there were effects on postintervention compared with preintervention on waist circumference. The analysis from the interviews showed that participants' motivation and barriers to continuing web‐based exercise vary widely and are influenced by personal, environmental and activity‐related factors. Those who continued exercising often experienced a positive impact on their health and well‐being and appreciated the social interaction and structure that exercise provided. Conversely, those who chose not to continue exercising, regardless of the type of exercise they continued with, experienced a lack of motivation, pain in their bodies or a lack of information on how they could continue exercising.

A systematic review by Sansano‐Nadal et al. (2019) measured the sustainability and long‐term effects of exercise interventions, focusing on whether participants continue to be physically active even after the intervention has formally ended. The target group was older adults, and the study presented similar results as the present study, with improvements observed for post‐intervention but no improvements for the long‐term follow‐up (Sansano‐Nadal et al. 2019). Nonetheless, even small improvements in physical activity are crucial for increasing PA at a population level and enhancing public health (Bull et al. 2020).

Research specifically focusing on web‐based exercise interventions is relatively new, and no consensus has been reached regarding their long‐term effectiveness, making the long‐term outcomes less certain (Van Biesen et al. 2024; Lang et al. 2022). Individuals with ID need more support when implementing new routines and motivation to enhance PA (Michalsen et al. 2020). This may help explain why existing research has reported challenges in sustaining exercise over the long term. Maybe the 12‐week intervention period needs to be extended for vulnerable groups in society, such as people with ID, to establish a sustainable exercise routine and effectively observe long‐term changes. A recent study by Hansen et al. (in press) explored the experiences of participants from the initial study by Fjellstrom et al. (2022) after the 12‐week programme, where several participants described feeling ‘stuck on the couch’ prior to the intervention. Their reliance on others, often support professionals, for planning, assistance and transportation to engage in physical activity reinforced this feeling. This underscores the critical role of staff support, a finding that was also highlighted in the present study.

The MVPA level observed in the present study was relatively high compared with other previous studies (Doody and Doody 2012; Jacinto et al. 2021), and a reason for this might be the difficulties in self‐reporting PA and the tendency to overestimate the amount of time being physically active (Ekelund et al. 2006). The reliability of the instrument used to analyse physical activity (IPAQ‐SF) may also be subject to scrutiny, particularly in assessing the long‐term effects of physical activity levels (Ekelund et al. 2006). Accordingly, these results should be interpreted with caution.

By implementing a web‐based exercise programme for people with ID, some of the barriers to PA can be avoided, such as transport and exposure to new environments (Bossink et al. 2017). This goes in line with the PAD model and how to maintain PA for the target group. By focusing on personalised interventions, supportive environments and effective behavioural strategies, the model can help maintain physical activity levels even after the formal intervention period ends. The fact that 41% of the participants continued with other forms of physical activity and 41% with web‐based exercise suggests that these individuals have successfully integrated physical activity into their daily lives. This indicates a high level of motivation and adaptability, which are crucial for the maintenance stage. However, the 18% who did not continue any physical activity underscore the challenges faced in maintaining motivation and integrating exercise into daily routines once the structured support of the intervention is removed. The varying percentages of sustained PA suggest differences in individual motivation and self‐efficacy. Those who continued with any form of exercise were likely to have higher intrinsic motivation or better self‐management skills. Additionally, access to resources and support persons likely influenced the ability to continue with physical activity (van der Ploeg et al. 2004). Interestingly, from the interviews, it became apparent that some participants were unaware they could continue with the web‐based exercise programme. Specifically, 18% of the participants who discontinued physical activity did not receive the support staff's inquiry regarding their interest in continuing with web‐based exercise after the intervention ended. Studies suggest that the influence of health professionals is likely more important for people with ID than for the general population, underscoring the importance of the support staff's engagement and involvement (van der Ploeg et al. 2004; Humpel et al. 2002; Fjellstrom et al. 2024). Staff training is also essential for implementation feasibility. Having qualified and trained staff to guide participants through the programme and provide support and motivation is critical for its success (Laxton et al. 2023). However, challenges related to staff training and turnover can impact programme continuity and quality over time. To overcome these challenges and enhance the implementation feasibility and scalability of web‐based exercise programmes for individuals with ID, a comprehensive approach is necessary. Moreover, continuous evaluation and adaptation of the programme based on participant and staff feedback are essential to ensure its effectiveness and long‐term sustainability.

There are several limitations to consider with the present study. First, the individuals who participated may have been the most motivated and engaged, which introduces a potential selection bias. Consequently, it remains unclear whether the nonparticipants (*n* = 10) continued exercising or their reasons for doing so. Second, the level of PA was self‐reported; thus, the use of objective measures is recommended in future research to more accurately capture changes in PA behaviour. Third, the study did not control for participants' food or fluid intake prior to body composition measurements, which may have affected the reliability of these results. This limitation was largely due to the practical challenges of ensuring high‐quality control in everyday settings, especially considering the participants' disabilities and the need to respect their privacy. Nonetheless, many individuals with ID often follow structured daily routines, which may have contributed to relatively consistent conditions across pre‐ and post‐measurements. Selecting appropriate measurement methods for this target group is challenging, as they must be feasible and not overly burdensome for ethical reasons. Strengths of the study include its mixed‐method design, which provided both a comprehensive understanding of the quantitative outcomes and the underlying experiences. Furthermore, this study is pioneering in its long‐term follow‐up of web‐based training, highlighting the need for more research in this field.

## 5 Conclusion

In conclusion, the findings from this study shed light on the long‐term effects of a web‐based exercise programme for individuals with ID. While significant changes were not observed after a 12‐month follow‐up, postintervention effects on waist circumference were noted. The study underscores the variability in participants' motivation and barriers to continuing web‐based exercise, influenced by personal, environmental and activity‐related factors. Notably, participants who continued exercising reported positive impacts on health and well‐being, emphasising the importance of structured support and social interaction. Conversely, those who ceased participation cited a lack of motivation and technical barriers, suggesting the need for tailored interventions and ongoing support. A web‐based exercise programme offers potential advantages in improving PA for people with ID and can help prevent barriers to increased physical activity for the target group. Moreover, collaboration among stakeholders and continuous programme evaluation is crucial for successful implementation and long‐term sustainability. Such efforts are likely to improve health outcomes for individuals with ID and enhance their overall well‐being.

## Funding

The study was funded by the Mid Sweden University agreement with the Municipality of Östersund.

## Ethics Statement

The study received preapproval from the Swedish Ethical Review Authority (Dnr 2019‐06495 and Dnr 2020‐02607). All research participants provided informed consent before participating in the study, with the understanding that their data would be presented anonymously and in aggregate form. No external materials have been included in this study.

## Consent

All participants provided explicit consent for the publication of the data collected during this study. They were informed that their data would be anonymised and presented using aggregated mean values to ensure their privacy and confidentiality.

## Conflicts of Interest

The authors declare no conflicts of interest.

## Data Availability

The data supporting the findings of this study can be obtained by contacting the corresponding author upon request. These data are not publicly available due to privacy or ethical restrictions.

## References

[jir70082-bib-0001] Ballin, M. , A. Hult , S. Bjork , J. Dinsmore , P. Nordstrom , and A. Nordstrom . 2020. “Digital Exercise Interventions for Improving Measures of Central Obesity: A Systematic Review.” International Journal of Public Health 65, no. 5: 593–605. 32410008 10.1007/s00038-020-01385-4 PMC7224590

[jir70082-bib-0002] Bossink, L. W. M. , A. A. van der Putten , and C. Vlaskamp . 2017. “Understanding Low Levels of Physical Activity in People With Intellectual Disabilities: A Systematic Review to Identify Barriers and Facilitators.” Research in Developmental Disabilities 68: 95–110. 28750208 10.1016/j.ridd.2017.06.008

[jir70082-bib-0003] Bull, F. C. , S. S. Al‐Ansari , S. Biddle , et al. 2020. “World Health Organization 2020 Guidelines on Physical Activity and Sedentary Behaviour.” British Journal of Sports Medicine 54, no. 24: 1451–1462. 33239350 10.1136/bjsports-2020-102955 PMC7719906

[jir70082-bib-0004] Carlson, L. 2013. “Research Ethics and Intellectual Disability: Broadening the Debates.” Yale Journal of Biology and Medicine 86, no. 3: 303–314. 24058305 PMC3767215

[jir70082-bib-0005] Castro, O. , K. Ng , E. Novoradovskaya , G. Bosselut , and M. Hassandra . 2018. “A Scoping Review on Interventions to Promote Physical Activity Among Adults With Disabilities.” Disability and Health Journal 11, no. 2: 174–183. 29132847 10.1016/j.dhjo.2017.10.013

[jir70082-bib-0006] Centers for Disease Control and Prevention . 2020. “Physical Activity and Health.” https://www.cdc.gov/physicalactivity/basics/pa‐health/index.htm.

[jir70082-bib-0007] Cleland, C. , S. Ferguson , G. Ellis , and R. F. Hunter . 2018. “Validity of the International Physical Activity Questionnaire (IPAQ) for Assessing Moderate‐to‐Vigorous Physical Activity and Sedentary Behaviour of Older Adults in the United Kingdom.” BMC Medical Research Methodology 18, no. 1: 176. 30577770 10.1186/s12874-018-0642-3 PMC6303992

[jir70082-bib-0009] Doody, C. M. , and O. Doody . 2012. “Health Promotion for People With Intellectual Disability and Obesity.” British Journal of Nursing 21, no. 8: 460. 22585073 10.12968/bjon.2012.21.8.460

[jir70082-bib-0010] Dumith, S. C. , P. C. Hallal , R. S. Reis , and H. W. Kohl 3rd. 2011. “Worldwide Prevalence of Physical Inactivity and Its Association With Human Development Index in 76 Countries.” Preventive Medicine 53: 24–28. 21371494 10.1016/j.ypmed.2011.02.017

[jir70082-bib-0011] Ekelund, U. , H. Sepp , S. Brage , et al. 2006. “Criterion‐Related Validity of the Last 7‐Day, Short Form of the International Physical Activity Questionnaire in Swedish Adults.” Public Health Nutrition 9, no. 2: 258–265. 16571181 10.1079/phn2005840

[jir70082-bib-0012] Elinder, L. S. , H. Bergstrom , J. Hagberg , U. Wihlman , and M. Hagstromer . 2010. “Promoting a Healthy Diet and Physical Activity in Adults With Intellectual Disabilities Living in Community Residences: Design and Evaluation of a Cluster‐Randomized Intervention.” BMC Public Health 10: 761. 21144033 10.1186/1471-2458-10-761 PMC3020685

[jir70082-bib-0013] Emerson, E. , C. Hatton , S. Baines , and J. Robertson . 2016. “The Physical Health of British Adults With Intellectual Disability: Cross Sectional Study.” International Journal for Equity in Health 15: 11. 26791808 10.1186/s12939-016-0296-x PMC4719222

[jir70082-bib-0014] Fjellstrom, S. , E. Hansen , J. Hollta , M. Zingmark , A. Nordstrom , and O. M. Lund . 2022. “Web‐Based Training Intervention to Increase Physical Activity Level and Improve Health for Adults With Intellectual Disability.” Journal of Intellectual Disability Research 66: 967–977. 36217301 10.1111/jir.12984 PMC9828805

[jir70082-bib-0015] Fjellstrom, S. , J. Holtta , A. Nordstrom , E. Flygare Wallen , M. Lund Ohlsson , and E. Hansen . 2024. “Increasing Physical Activity Through an Adapted Web‐Based Exercise Program for People With Intellectual Disabilities: Support Staff Are Crucial for Feasibility.” Journal of Applied Research in Intellectual Disabilities 37, no. 2: e13191. 38369314 10.1111/jar.13191

[jir70082-bib-0016] Graneheim, U. H. , B. M. Lindgren , and B. Lundman . 2017. “Methodological Challenges in Qualitative Content Analysis: A Discussion Paper.” Nurse Education Today 56: 29–34. 28651100 10.1016/j.nedt.2017.06.002

[jir70082-bib-0017] Graneheim, U. H. , and B. Lundman . 2004. “Qualitative Content Analysis in Nursing Research: Concepts, Procedures and Measures to Achieve Trustworthiness.” Nurse Education Today 24, no. 2: 105–112. 14769454 10.1016/j.nedt.2003.10.001

[jir70082-bib-0018] Hansen, E. , J. Hölttä , S. Fjellström , E. Flygare Wallén , M. Jong , and L. Ohlsson . 2023. I Am Able, Happier, Livelier and Stronger—Perceptions of Adults With Intellectual Disability Participating in a Web‐Based Physical Activity Intervention [Manuscript submitted for publication]. Department of Health Sciences. Mid Sweden University.

[jir70082-bib-0019] Humpel, N. , N. Owen , and E. Leslie . 2002. “Environmental Factors Associated With Adults' Participation in Physical Activity: A Review.” American Journal of Preventive Medicine 22, no. 3: 188–199. 11897464 10.1016/s0749-3797(01)00426-3

[jir70082-bib-0020] Jacinto, M. , D. Monteiro , R. Antunes , J. P. Ferreira , R. Matos , and M. J. Campos . 2023. “Effects of Exercise on Body Mass Index and Waist Circumference of Individuals With Intellectual and Developmental Disabilities: A Systematic Review With Meta‐Analysis.” Frontiers in Physiology 14: 1236379. 37601630 10.3389/fphys.2023.1236379 PMC10433222

[jir70082-bib-0021] Jacinto, M. , A. S. Vitorini , D. Palmeira , et al. 2021. “Percieved Barriers of Physical Activity Participation in Individuals with Intellectual Disability ‐ A Systematic Review.” Healthcare 9, no. 11: 1521. 10.3390/healthcare9111521. 34828567 PMC8625076

[jir70082-bib-0022] Jacob, U. S. , J. Pillay , E. Johnson , O. T. Omoya , and A. P. Adedokun . 2023. “A Systematic Review of Physical Activity: Benefits and Needs for Maintenance of Quality of Life Among Adults With Intellectual Disability.” Frontiers in Sports and Active Living 5: 1184946. 37361407 10.3389/fspor.2023.1184946 PMC10285488

[jir70082-bib-0023] Katzmarzyk, P. T. , C. Friedenreich , E. J. Shiroma , and I. M. Lee . 2022. “Physical Inactivity and Non‐Communicable Disease Burden in Low‐Income, Middle‐Income and High‐Income Countries.” British Journal of Sports Medicine 56, no. 2: 101–106. 33782046 10.1136/bjsports-2020-103640 PMC8478970

[jir70082-bib-0024] Kurtze, N. , V. Rangul , and B. E. Hustvedt . 2008. “Reliability and Validity of the International Physical Activity Questionnaire in the Nord‐Trondelag Health Study (HUNT) Population of Men.” BMC Medical Research Methodology 8: 63. 18844976 10.1186/1471-2288-8-63 PMC2577099

[jir70082-bib-0025] Lang, S. , C. McLelland , D. MacDonald , and D. F. Hamilton . 2022. “Do Digital Interventions Increase Adherence to Home Exercise Rehabilitation? A Systematic Review of Randomised Controlled Trials.” Archives of Physiotherapy. 12, no. 1: 24. 36184611 10.1186/s40945-022-00148-z PMC9527092

[jir70082-bib-0026] Laxton, P. , F. Patterson , and S. Healy . 2023. “Factors Related to Physical Activity in Adults With Intellectual Disabilities in Group Home Settings: A Systematic Literature Review.” Adapted Physical Activity Quarterly 40, no. 2: 347–377. 36543174 10.1123/apaq.2022-0064

[jir70082-bib-0027] Lee, I. M. , E. J. Shiroma , F. Lobelo , et al. 2012. “Effect of Physical Inactivity on Major Non‐Communicable Diseases Worldwide: An Analysis of Burden of Disease and Life Expectancy.” Lancet 380, no. 9838: 219–229. 22818936 10.1016/S0140-6736(12)61031-9 PMC3645500

[jir70082-bib-0029] Lotan, M. , S. Yalon‐Chamovitz , and P. L. Weiss . 2010. “Virtual Reality as Means to Improve Physical Fitness of Individuals at a Severe Level of Intellectual and Developmental Disability.” Research in Developmental Disabilities 31, no. 4: 869–874. 10.1016/j.ridd.2010.01.010. 20346616

[jir70082-bib-0030] McKeon, M. , E. Slevin , and L. Taggart . 2013. “A Pilot Survey of Physical Activity in Men With an Intellectual Disability.” Journal of Intellectual Disabilities 17, no. 2: 157–167. 10.1177/1744629513484666. 23539608

[jir70082-bib-0031] Michalsen, H. , S. C. Wangberg , A. Anke , G. Hartvigsen , L. Jaccheri , and C. Arntzen . 2020. “Family Members and Health Care Workers' Perspectives on Motivational Factors of Participation in Physical Activity for People With Intellectual Disability: A Qualitative Study.” Journal of Intellectual Disability Research 64: 259–270. 31981261 10.1111/jir.12716

[jir70082-bib-0032] Park, K.‐H. , J.‐S. Kim , S. Kim , and B. Ku . 2024. “A Systematic Review of Web‐Based Physical Activity Interventions for Individuals With Disabilities.” IJASS (International Journal of Applied Sports Sciences) 36: 77–90. 10.24985/ijass.2024.36.1.77.

[jir70082-bib-0033] Ross, R. , I. J. Neeland , S. Yamashita , et al. 2020. “Waist Circumference as a Vital Sign in Clinical Practice: A Consensus Statement From the IAS and ICCR Working Group on Visceral Obesity.” Nature Reviews. Endocrinology 16, no. 3: 177–189. 10.1038/s41574-019-0310-7 PMC7027970 32020062

[jir70082-bib-0034] Sansano‐Nadal, O. , M. Gine‐Garriga , J. S. Brach , et al. 2019. “Exercise‐Based Interventions to Enhance Long‐Term Sustainability of Physical Activity in Older Adults: A Systematic Review and Meta‐Analysis of Randomized Clinical Trials.” International Journal of Environmental Research and Public Health 16, no. 14: 2527. 31311165 10.3390/ijerph16142527 PMC6678490

[jir70082-bib-0035] Schalock, R. L. , R. Luckasson , and M. J. Tasse . 2021. “An Overview of Intellectual Disability: Definition, Diagnosis, Classification, and Systems of Supports (12th ed.).” American Journal on Intellectual and Developmental Disabilities 126, no. 6: 439–442. 34700345 10.1352/1944-7558-126.6.439

[jir70082-bib-0036] Van Biesen, D. , T. Van Damme , N. Morgulec‐Adamowicz , A. Buchholz , M. Anjum , and S. Healy . 2024. “A Systematic Review of Digital Interventions to Promote Physical Activity in People With Intellectual Disabilities and/or Autism.” Adapted Physical Activity Quarterly 41, no. 2: 330–350. 37793654 10.1123/apaq.2023-0061

[jir70082-bib-0037] van der Ploeg, H. P. , A. J. van der Beek , L. H. van der Woude , and W. van Mechelen . 2004. “Physical Activity for People With a Disability: A Conceptual Model.” Sports Medicine 34, no. 10: 639–649. 15335241 10.2165/00007256-200434100-00002

[jir70082-bib-0038] Vikberg, S. , N. Sorlen , L. Branden , et al. 2019. “Effects of Resistance Training on Functional Strength and Muscle Mass in 70‐Year‐Old Individuals With Pre‐Sarcopenia: A Randomized Controlled Trial.” Journal of the American Medical Directors Association 20, no. 1: 28–34. 30414822 10.1016/j.jamda.2018.09.011

[jir70082-bib-0039] World Health Organization . 2001. International Classification of Functioning, Disability and Health (ICF). World Health Organization.

[jir70082-bib-0040] Yang, W. , X. Liang , and C. H. Sit . 2022. “Physical Activity and Mental Health in Children and Adolescents With Intellectual Disabilities: A Meta‐Analysis Using the RE‐AIM Framework.” International Journal of Behavioral Nutrition and Physical Activity 19, no. 1: 80. 35799257 10.1186/s12966-022-01312-1 PMC9261031

